# Cytokines: Key Determinants of Resistance or Disease Progression in Visceral Leishmaniasis: Opportunities for Novel Diagnostics and Immunotherapy

**DOI:** 10.3389/fimmu.2019.00670

**Published:** 2019-04-05

**Authors:** Alti Dayakar, Sambamurthy Chandrasekaran, Suresh V. Kuchipudi, Suresh K. Kalangi

**Affiliations:** ^1^Independent Researcher, Vizianagaram, India; ^2^Bio5 Institute, University of Arizona, Tucson, AZ, United States; ^3^Animal Diagnostic Laboratory, Department of Veterinary and Biomedical Sciences, The Pennsylvania State University, University Park, PA, United States; ^4^Department of Biosciences, School of Sciences, Indrashil University, Mehsana, India

**Keywords:** *Leishmania*, cytokines, T-cells, visceral leishmaniasis, diagnosis, immunotherapy

## Abstract

Leishmaniasis is a parasitic disease of humans, highly prevalent in parts of the tropics, subtropics, and southern Europe. The disease mainly occurs in three different clinical forms namely cutaneous, mucocutaneous, and visceral leishmaniasis (VL). The VL affects several internal organs and is the deadliest form of the disease. Epidemiology and clinical manifestations of VL are variable based on the vector, parasite (e.g., species, strains, and antigen diversity), host (e.g., genetic background, nutrition, diversity in antigen presentation and immunity) and the environment (e.g., temperature, humidity, and hygiene). Chemotherapy of VL is limited to a few drugs which is expensive and associated with profound toxicity, and could become ineffective due to the parasites developing resistance. Till date, there are no licensed vaccines for humans against leishmaniasis. Recently, immunotherapy has become an attractive strategy as it is cost-effective, causes limited side-effects and do not suffer from the downside of pathogens developing resistance. Among various immunotherapeutic approaches, cytokines (produced by helper T-lymphocytes) based immunotherapy has received great attention especially for drug refractive cases of human VL. Therefore, a comprehensive knowledge on the molecular interactions of immune cells or components and on cytokines interplay in the host defense or pathogenesis is important to determine appropriate immunotherapies for leishmaniasis. Here, we summarized the current understanding of a wide-spectrum of cytokines and their interaction with immune cells that determine the clinical outcome of leishmaniasis. We have also highlighted opportunities for the development of novel diagnostics and intervention therapies for VL.

## Introduction

Leishmaniasis is a neglected tropical disease (NTD) caused by an obligatory intracellular protozoan parasite that belongs to the genus *Leishmania*. It is a vector-borne infection transmitted by female sandflies and the disease is highly prevalent in poor and malnourished populations of the world living in tropical and subtropical countries. The life cycle of *Leishmania* is simple and the parasite propagates in two different morphological forms. The promastigote stage of the parasite exists in the insect body fluids and enters the mammalian host when sandfly takes a blood meal. Promastigotes transform into amastigotes inside the mononuclear phagocytes of hosts and establish the infection by evading host defense system (1). The infected individuals could develop self-healing cutaneous ulcers to life-threatening visceral disease (2). World-wide, 0.7–1.0 million new cases of leishmaniasis and 20,000 to 30,000 deaths are reported each year (3). Visceral leishmaniasis (VL) or kala-azar is the deadliest clincial form of leishmaniasis, typically caused by *L. donovani* and *L. infantum* in the Old World and *L. chagasi* in the New World. Occasionally, *L. tropica* and *L. amazonensis* have also been found to cause VL in the Middle East and South America, respectively (4). The anthroponotic transmission of VL is prevalent in the Indian subcontinent (5). The annual report of global VL indicates that there are 50,000 to 90,000 new cases each year with high incidence in the Indian subcontinent and East Africa (3). VL is an opportunistic infection and has been identified as a co-infection in HIV patients (6). HIV infection amplifies the risk of developing active VL and the severity by 100–2,320 times (7). Fever, weight-loss, anemia, pancytopenia, hyperpigmentaion of skin and hepatosplenomegaly are some of the manifestations of VL and the mortality rate is over 95% (3). Children under the age of 1 year and adults above 50 years of age are highly susceptible to VL (8, 9). The susceptible host genetic background (10), nutritional status especially malnutrition (11) and immune suppression (12) ameliorates the clinical outcome of the disease. The current VL treatment relies mostly on chemical drugs like pentavalent antimonials (Sb^V^), amphotercin B, miltefosine, and paromomycin etc. But their misuse, life-threatening toxicity, and development of resistance by the parasites (13) highlight the need for drug-sparing alternative therapeutic strategy to combat the clinical disease. Recently, immunotherapy has emerged as a promising option to control various diseases including VL. This review presents an in-depth critical analysis of immune responses to leishmaniasis and highlights prospective cytokine candidates that could be used for the diagnosis and therapy of VL.

### *Leishmania* Infection and Innate Immune Cells

*Leishmania* infection in humans is usually subclinical and parasites may persist for life-time of the host through several escape mechanisms (14). For example, *Leishmania* blocks the maturation of complement system and C_5_–C_9_ membrane attacking complex formation, reduces the expression of B7 and CD40 that are required for T-cell anti-parasitic activity, promote overexpression of the iron transporters, modifies the toll-like receptor (TLR)-2/TLR-4 signaling and inhibits Janus tyrosine kinase/signal transducer and activator of transcription (JAK/STAT) pathway in macrophages (MΦs) thereby turnoff the cytokine cascade, and alters the expression profile of cytokines and chemokines etc. It is clear that *Leishmania* parasites manipulate several key aspects of host defense for their survival. Consequently, targeting immune components is a reliable method to combat the disease. In addition, host innate immune signatures that are specific to *Leishmania* infection could help early prediction of the disease outcome. These include aspects of innate immune response, such as front-line defense led by the natural killer (NK) cells, mononuclear and polymorphonuclear phagocytes (15). In general, *Leishmania* parasite resists their uptake by phagocytic dendritic cells (DCs) and MΦs (16, 17) by inhibiting reactive oxygen species (ROS) production that delays phagolysosome formation (18) and blocks lysosomal proteolytic degradation (19). The complement protein C3b, a potent immune opsonin accelerates phagocytosis of *Leishmania* (17) by interacting with the parasite surface glycoprotein gp63 (20). MΦs and DCs that engulfed *Leishmania* activate their TLR-9 signaling and produce interleukin (IL)-12, which stimulates NK cells to produce interferon (IFN)-γ, a key cytokine that is responsible for skweing Th1 response (16, 21) and stimulate the MΦs to produce ROS and nitric oxide (NO) for oxidative killing of intracellular amastigotes thereby protects the host (22–25). To establish an early infection, *L. major* inhibits the NK cell proliferation and IFN-γ production (26) and *L. donovani* evades inducible nitric oxide synthase (iNOS)-dependent killing of intracellular amastigotes in MΦs via downregulation of iNOS mRNA expression (27, 28) and induction of arginase expression (29) as the arginine is a common substrate for both iNOS and arginase enzymes. Thus, MΦs play a complex role in *Leishmania* pathogenesis and are associated with both survival and death of the parasites (30). Further, the induction of FasL-mediated apoptosis in *Leishmania* infected MΦs is a part of host defense mechanisms in innate immunity (31). Similarly, other immune cells also play a key part in the early host defense against leishmaniasis. For example, a drastic reduction in IL-8 and eotaxin secretion from neutrophils and eosinophils, respectively (32), and an elevated number of IL-4^+^ neutrophils and IL-10^+^ eosinophils and reduced number of IFN-γ^+^ and IL-12^+^ eosinophils are observed in active VL patients (33).

### Origin of Th1-Th2 Dichotomy in Leishmaniasis

**Figure 1:**
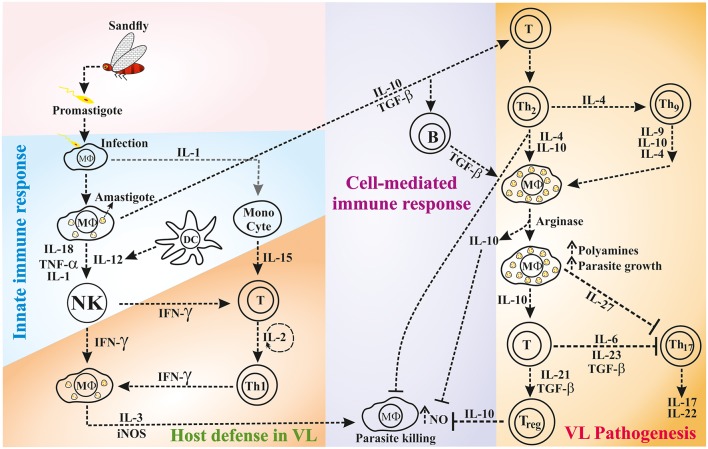
The interaction of innate and/or adaptive immune cells via cytokines during host defense or disease progression in VL.

While the host innate immune response against leishmaniasis is important, it is now clear that the T-cell mediated immunity and the cytokines produced from various immune cells play a crucial role in determining the disease outcome (shown in Figure 1). However, the cytokines function in autocrine (locally) and paracrine (at a distance from the site of synthesis) fashion to regulate the immune response (34). A longitudinal study on *Leishmania* pathogenesis and disease recovery highlighted the role of helper T (Th)-cell responses (35). Therefore, immune cells and their cytokines have been recognized as potential targets for immunotherapy to modulate the activity of factors that are crucial in the immune system for healing. In this context, the phenomenon called “Th1-Th2 dichotomy” became popular based on the role of the cytokines produced by these cells in disease progression and/or host protection. Mosmann et al. reported for the first time that the cloned murine Th-cells are in two functional subsets namely Th1 and Th2 based on the production of IFN-γ and IL-4, respectively (36). Thereafter, several studies demonstrated the key role of major cytokines [e.g., IL-10, Transforming growth factor (TGF)-β, IL-4, IL-6, IL-12, and IFN-γ] that implicated the role of Th1/Th2 balance in disease progression or host protection. In general, Th1 type response mediates host resistance and Th2 type response associates with disease progression (37). In resistant mouse strains, the abundance of Th1 type cytokines; IFN-γ, IL-2, and lymphotoxin spontaneously cleared the *L. major* infection, whereas, in susceptible mouse strains, infection led to the fatal disease by the action of Th2 type cytokines; IL-4, IL-5 and IL-10 (38). IL-4 and IL-10 associated with the visceralization of cutaneous *L. major* infection (39, 40). However, the Th1-Th2 dichotomy is more complex than previously recognized, which is more evident in certain cases of leishmaniasis (41), such as *L. donovani* infection where the susceptibility of mouse strains is variable (42). Unlike in cutaneous leishmaniasis (CL), T-cells with Th2 phenotype are difficult to demonstrate in the mouse model of VL (37, 40–43). Similarly, the association between Th1 response and disease resistance to VL is complex in humans (44, 45). Occasionally, individuals respond to the exposure of *Leishmania* antigens via T-cells even they have no prior exposure to the parasite; this is possible due to the cross-activity by other microorganisms (46).

### Immune Response During VL

Immune response in VL patients is characterized by abundant anti-leishmanial antibody titers and low or absence of *Leishmania*-specific T-cell proliferation and IL-2 and IFN-γ production. Recovery from VL is mostly dependent on the induction of T-cell immunity; preferably Th1 response, which is primed by IL-12^+^ DCs and MΦs (47, 48). IFN-γ produced from IL-12 primed T-cells induce NO-mediated killing of the parasites (49, 50). In contrast, VL progression in humans is associated with abundant production of Th2 type cytokines IL-10, TGF-β, and IL-4 or presence of IL-10^+^ regulatory T cells (Tregs), which diminish the anti-parasitic activity of M1-type MΦs and Th1 response (51–53). However, the presence of abundant IL-10 is crucial rather than a lack of IFN-γ in the VL clinical disease progression (54). IL-10 partially inhibits IFN-γ production but strongly resists IFN-γ mediated activation of MΦs while killing the intracellular parasites (55–57). Likewise, the lack of IFN-γ may result in relatively higher levels of IL-10 in human leishmaniasis resulting in MΦ deactivation (58) and parasite proliferation (59). Murine model of VL demonstrates higher disease susceptibility due to the presence of high IL-10 levels during initial phase of infection (60). The splenic infection of *L. donovani* causes a constitutive expression of chemokine ligand 2 (CCL2) or monocyte chemoattractant protein 1 (MCP-1), which triggers IL-4 secretion from Th2 cells that activates the MΦs in alternative manner. These M2-type MΦs express arginase in abundant quantity and help in the biosynthesis of polyamines, which favor the survival and growth of the parasite (61). During chronic VL, the high expression of programmeded death protien-1 (PD-1) or Cytotoxic T-lymphocyte Antigen 4 (CTLA-4) causes unresponsiveness in CD4^+^ T-cell, which produce TGF-β in abundant levels and helps in persistence of infection (62). Taken together, there is mounting evidence suggesting that Th1-Th2 imbalance and T-cell unresponsiveness are critical issues in VL pathogenesis.

### Objective of the Review

**Figure 2:**
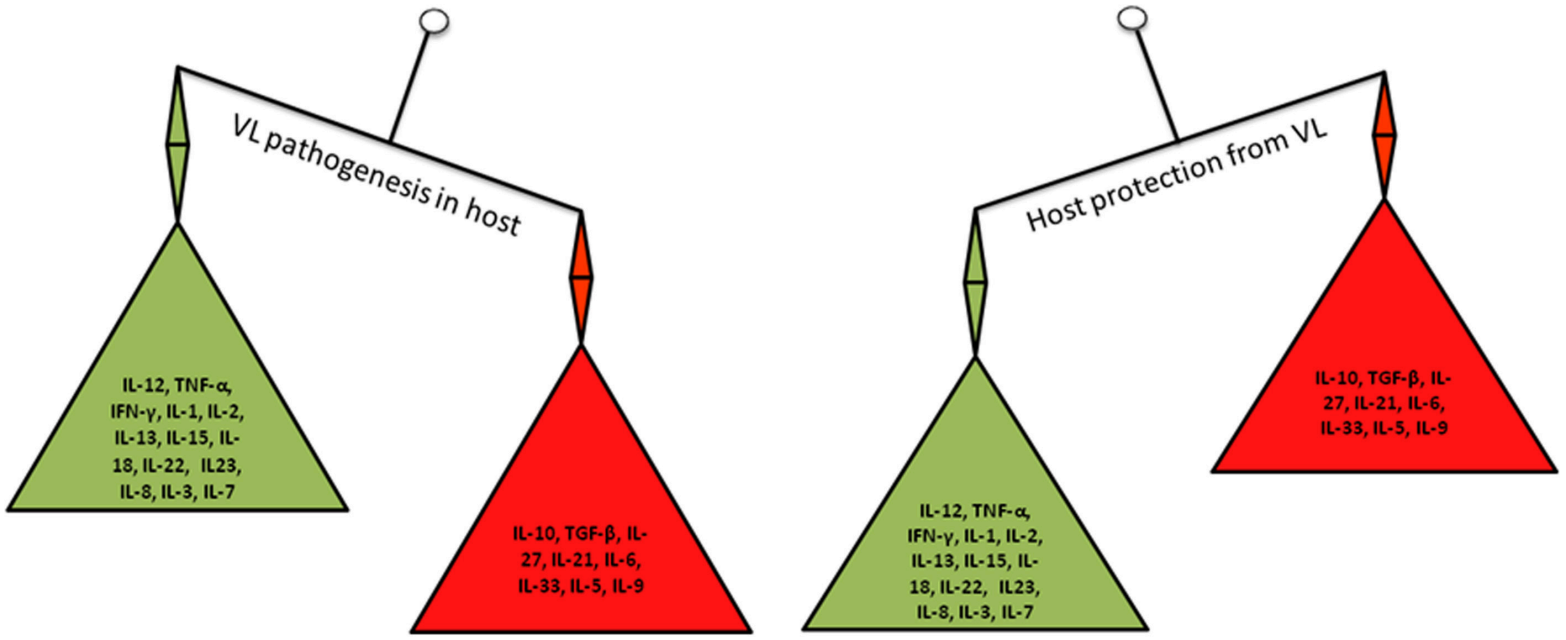
Cytokines balance in VL pathogenesis and host protection.

Role of a whole host cytokines in the resistance and disease progression during VL is increasingly being uncovered. Till date targeting either Th1 or Th2 cytokines produced promising results for leishmaniasis cure. Th1/Th2 balance is not the only determinant of the outcome of leishmaniasis as previously thought because a range of other cytokines have recently been implicated in both disease progression and host protection (Figure 2). Hence, there is a need for in-depth analysis of the role of cytokines and *Leishmania* pathogenesis to get a comprehensive view of the complex interplay of *Leishmania* parasite and their hosts. This review aims to summarize and critically analyze the state-of-the-art knowledge relating to cytokines and VL pathogenesis. Special emphasis has been made for the identification of potential cytokine targets that could be used for the development of novel diagnostic assays and immunotherapies for the detection and treatment of VL.

## Cytokine Response in Leishmaniasis

It is well-known that cytokines play a role in pathogenesis and hosts resistance of VL. Cytokines that play crucial role in *Leishmania* pathogenesis or host defense are tabulated in Tables 1, 2, respectively. However, there are several cytokines that are play a dual role both in the disease progression and host resistance are summarized in Table 3. Cytokines targets for diagnosis and/or immunotherapy are shown in Table 4. Functions of individual cytokines as relates to pathogenesis and/or host resistance are discussed in detail in the sections below.

**Table 1:** Cytokines involve in the host protection.

**Cytokine**	**Relative functions in leishmaniasis**	**References**
IFN-γ	Activates MΦs and monocytes to release oxygen radicals and TNF-α, IL-l, and IL-6 secretion Blocks the production of IL-10	(63–67)
	Absence leads to Th2 skewing	(48)
IL-12	Drives Th1 response and IFN-γ production	(68)
	Controls Th2 expansion and IL-4 production	(69, 70)
	Induces NOS2 expression and NO production	(71)
	Induces cell proliferation and lymphokines production	(71, 72)
TNF-α	Activates the MΦs to kill amastigotes	(73)
	Induces NO production to kill the parasite or inhibit visceralization	(74)
	Induces granuloma response and wound healing process	(75, 76)
	Shows IFN-γ-independent leishmanicidal activity	(77, 199)
	Promotes IL-10 producing T-cells for immune homeostasis	(78)
IL-2	Activates T-cells and NK cells and induces IFN-γ production	(79, 80)
	Induces the production of IL-4	(81)
	Endogenous IL-2 shows host protection	(82)
	Exogenous IL-2 exerts anti-leishmanial action even in the absence of IFN-γ	(83)
IL-15	Synergizes with IL-2 and IL-12 functions	(84, 85)
	Induces T-cell proliferation and inhibits apoptosis, preserves memory T-cells, and induces B-cell maturation and isotype switching	(86–88)
	Activates both Th1 and Th2 subtypes and shows pleiotropic role	(89, 90)
	Stimulates Th1 response, IL-12 production and downregulates IL-4+ Th2 cells	(86, 91)
IL-22	Promotes inflammatory response and is crucial in tissue repair	(92, 93)
	Protects the liver from chronic infections	(94)
	Induces the production of antimicrobial peptide-β-defensin	(95)
	Complementary to Th1 cytokines and requires IL-6 for production	(96–98)
IL-7	Induces proliferation of thymocytes, NK and mature T-cells, and production of cytotoxic T-cells	(99–104)
	Promotes the synthesis and secretion of IL-6, TNF-α, IL-1α, IL-1β, and MIP-113 by monocytes	
	With the combination of IFN-γ, it induces TNF-α and NO production to kill the amastigotes	(105)
IL-8	Promotes the recruitment of neutrophils and granulocytes at lesion site	(106, 107)
	Declines in the serum of active VL and polymorphism at −251 position associates with active VL	(32, 108)
IL-23	Shows IL-12 independent protection against visceral infection	(76, 109, 110)
	P19 pairs with IL-12p40 to become active and protects the host	(111)

**Table 2:** Cytokines involve in the disease progression.

**Cytokine**	**Relative functions in leishmaniasis**	**References**
IL-10	Inhibits IFN-γ, IL-1, IL-6, IL-12, and TNF-α production and downregulates the innate and T-cell specific immunity	(112, 113)
	Downregulates Th1 response, MΦ activation and DC's antigen presentation	(58, 114)
	Inhibits reactive nitrogen intermediates and IFN-γ production	(115)
	Protects the tissues from inflammatory damage	(116)
	Inhibits acute inflammation required for the parasite clearance	(117)
TGF-β	Inhibits T-cell proliferation, MΦ activation, iNOS expression, TNF-α and IFN-γ functions	(118)
	Shows marginal effect on the parasite load and IFN-γ dependent host resistance	(119)
	Modulates immune response in favor of the parasites growth	(120)
	Enhances arginase expression and polyamines synthesis	(121–123)
	Shows the biphasic kinetics; promotes as well as inhibits the inflammation	(62, 124)
	Impairs the rate of disease cure in murine models	(125, 126)
IL-5	Higher production at lesion site and declines Th1 polarization	(127, 128)
	Favors the parasite growth and dissemination	(129)
IL-6	Induces differentiation of monocytes from DCs to MΦs	(130)
	Favors Th2 response and suppresses MΦ activation	(75)
	Endogenous IL-6 shows host suppressive role in *L. donovani* infection	
	Inhibits IFN-γ mediated gene expression	(131)
	Absence of signaling induces Th1 response, tissue inflammation and parasite killing	(132)
	Induces IL-27 and IL-10 production in *L. donovani* infection model	(54, 133–135)
IL-9	Regulates Th1-Th2 balance and expresses in *L. major* susceptible mice even after 4-weeks but not in resistant mice	(136)
	Neutralization induces Th1 response and delays the disease progression	(137)
IL-27	Mediates anti-inflammatory response by suppressing Th17 cells	(138, 139)
	Induces T-bet expression and IL-10 secretion by autocrine action of IL-21 on CD4^+^ T-cells	(140, 141)
	IL-4 induced IL-6 and TGF-β inhibits IL-27 mediated Th1 response	(142)
	IL-27 is not required for Th1 development and induces IL-10 in *L. donovani* infection	(140, 143–145)
	Absence of signaling leads to Th1 response, tissue inflammation, and rapid parasite killing in *L. donovani* infection to liver	(71, 146)
	Elevates in human plasma and spleen during active VL	(140)
IL-33	ST2-expressing T-cells accumulates in lesion site and polyclonal anti-ST2 antiserum reduces Th2 response and lesion growth	(147)
	IL-33 is abundant in serum and IL33+ cells in liver of VL patients	(148)
	ST2^−/−^ induces IFN-γ and IL-12 and controls the parasite load in liver	
	rIL-33 reduces Th1 immunity and infiltration of PMNs and monocytes in liver	(149)

**Table 3:** Cytokines with dual role in leishmaniasis.

**Cytokine**	**Relative functions in leishmaniasis**	**References**
IL-4	Inhibits IFN-γ production and triggers alternative activation of MΦs and parasite survival	(150–152)
	Inhibits oxidative burst through reducing ROS and NO in MΦs	(153, 154)
	IL-4R expression is abundant in *L. major* infection	(155)
	Modulates antigen-uptake and endosomal processing, promotes humoral response in favor of disease	(156)
	Induces IFN-γ secretion from CD8^+^ T cells in *L. donovani* infection	(157)
	Produces from PBMCs of cured VL patients in response to *L. donovani* crude or purified gp63 antigen stimulation	(158)
IL-13	IL-13 knock-out depletes granuloma response and IFN-γ secretion, and enhances IL-4 and IL-10 production in *L. donovani* infection	(159)
	Protects from *L. major* infection via IL-1β and IL-12 production	(160)
	IL-4 and IL-13 involves in pathogenesis of *L. mexicana, L. amazonensis*, and *L. (V.) panamensis* infections	(161)
	Parasite species and the host genetic background influence the dual role of IL-13	(162)
IL-17	Induces TNF-α, IL-1, and chemokines production	(163)
	Affects neutrophils function, reduces apoptosis, induces the production of pro-inflammatory cytokines and tissue damaging molecules at inflammatory foci	(164, 165)
	Induces GM-CSF, G-CSF, CXCL8, CXCL1, CXCL6, and CXCL10	(96, 97)
	Induces IL-6 production and mediates pro-inflammatory and regulatory functions	(96)
	Complementary to Th1 response and protects from *L. donovani* infection but increases susceptibility for *L. major and L. (V.) braziliensis*	(166, 167)
IL-18	Drives Th1 and NK cell development and induces IFN-γ production	(168–170)
	Regulates the expansion of Th2 cells and stimulates TNF-α secretion by human PBMCs	(171, 172)
	In combination with IL-12, it activates memory cells and prevents reinfection of *L. major*	(173)
	Deficiency induces the susceptibility for *L. donovani* infection	(174)
	Induces IFN-γ independent protection from *L. donovani* infection and stimulates IL-4 and IL-13 production	(175, 176)
	IL-18^−^/^−^ increases the resistance to *L. mexicana* infection by inducing the secretion of IFN-γ and IgG2a and reducing IL-4, IgG1, and IgE	(177)
	Regulates Th1 and Th2 balance *in vivo*	(168)
IL-1	Induces inflammation and controls parasite dissemination	(178, 179)
	IL-1β coordinates immune-to-brain communication	
	IL-1α inhibits disease progression in *L. major* infected mice	(180)
	Deficiency of IL-1 family genes delays the disease progression in *L. major* infection	(178, 181)
	Low IL-1 induces the susceptibility for *L. donovani* infection	
	Impaired production of IL-1 from human PBMCs with *L. donovani*-antigen stimulation and successful therapy recovers IL-1 levels	(182–184)
	rIL-1α induces granuloma response and IFN-γ production but not able to clear the parasite	(185)
IL-3	With the combination of GM-CSF, M-CSF, and IFN-γ, it induces oxidative burst and TNF-α secretion and inhibits the parasite growth	(186)
	With M-CSF combination, it induces superoxide ions production and kills the parasites during acute VL	
	Provokes the infection in murine model of CL	

**Table 4:** Cytokines used in diagnosis and chemo/immunotherapy of VL.

**Cytokine**	**Relative functions in leishmaniasis**	**References**
Anti-IL-10R	Controls the experimental VL and induces antimonials activity in IL-10 knock-out or transgenic mice and IFN-γ production	(53, 56)
IL-10	Neutralization increases IFN-γ and TNF-α production and reduces parasite burden in VL patients	(187)
	Abundant in antigen-stimulated PBMCs of *L. chagasi* infection	
	In asymptomatic individuals, IL-10 not directly correlates with Montenegro test positivity	(188)
	Balanced IL-10 and IL-12 response induces chemotherapy efficacy	(17)
	Disease relapse in human VL associates with IL-10 and IL-10^+^IFN-γ^+^ antigen-specific T-cells	(189)
	Clinical symptoms strongly correlates with IL-6, IL-27, TNF-α, and IL-10 in *L. infantum* infected Brazilian population	(190)
IL-4	Upregulates in VL and associates with impaired treatment	(191, 192)
	IFN-γ, IL-4, and IL-13 certainly upregulates in active VL and declines after cure	(193)
IFN-γ	Absence of antigen-specific lymphocyte proliferation and IFN-γ production indicates clinical disease	(45)
	Useful in assessing candidacy of vaccine antigens	(194)
	Sb^v^ with rIFN-γ had shown 82.3, 75, and 87% efficacy against VL patients from Brazil, Kenya, and India, respectively	(195–197)
IL-12	Induces better response than anti-IL-10 alone or in combination with anti-IL-4 from PBMCs of VL patients	(115)
	VL cure restores the IFN-γ and IL-12 production	(48)
	Useful as effective adjuvant for a killed vaccine against *L. major*	(198)
	rIL-12 mediates the cure of *L. major* infection, induces Th1 cytokines and inhibits IL-4	(75, 81, 200)
	Neutralization exacerbates *L. major* and *L. donovani* infections	(49, 69, 199)
IL-15	Liposomal amphotericin-B induces plasma IL-15 levels in VL	(86)
	IL-15 with combination of IFN-γ or IL-12 increases efficacy of antimonial therapy for VL	(201)
TNF-α	Anti-TNF-α therapy for arthritis increases susceptibility to VL	(202, 203)
	In HIV co-infection, high levels of serum TNF-α and IFN-γ predicts the onset of acute VL	(204)
TGF-β & IL-13	Antagonists of these clears the VL marginally and had no synergy with Sb^V^	(119)

### IL-10 Is a Key Player in Disease Progression

IL-10 is an 18 kDa pleiotropic cytokine, primarily produced by alternatively activated MΦs, DCs, and lymphocytes. As an immunoregulatory cytokine, IL-10 exerts multiple biological effects on different cell types (205). IL-10 is the product of Th2 subset, also known as cytokine synthesis inhibitory factor (CSIF) since it suppresses IFN-γ production from Th1 cells (112). IL-10 is known to inhibit production of cytokines like IL-1, IL-6, IL-12, and tumor necrosis factor (TNF)-α. In addition, Il-10 also inhibits MΦ mediated activation of T-cell through the reduced expression of class II major histocompatibility complex (MHC) and co-stimulatory molecules on the surface of MΦ and results in the inhibition of both innate and T-cell mediated immunity (188). The suppressive role of IL-10 in human VL results in the drastic fall in accumulation of monocyte derived macrophages, which is regulated by migration inhibitory factor (MIF). Further, IL-10 plays a substantial role in the pathogenesis of leishmaniasis by causing the downregulation of Th1 response, MΦ activation (114) and antigen presentation by DCs (58). Furthermore, IL-10 inhibits the leishmanicidal functions of MΦ (206) by diminishing the production of reactive nitrogen intermediates by MΦ, IFN-γ by T and natural killer (NK) cells (115), and IL-12 mediated activation of MΦ (48). High levels of IL-10 during the initial phases of infection due to decreased multifunctional CD4 T cells results in higher susceptibility to VL. Despite elevated levels of IFN-γ during the steady state of infection, parasite burden is not reduced due to higher levels of IL-10 (60). The unfavorable clinical outcome in localized CL was correlated with IL-10 but not with inadequate Th1 response (207). High levels of serum IL-10 is associated with symptomatic VL but absent in asymptomatic individuals. A key function of IL-10 is to protect the tissues from collateral damage due to excessive inflammation (116). However, in the face of parasitic infection an acute inflammatory response is necessary to control the parasite proliferation, hence, the anti-inflammatory role of IL-10 may help the disease progression (117). During active VL, CD8^+^ T-cells could also play an important role in disease progression via abundant production of IL-10 (208). However, the role of IL-10 in VL appears to be species-specific as it was suggested that IL-10 may not be a regulatory cytokine in canine VL. In experimental CL, a group of Treg cells namely, CD4^+^CD25^+^Foxp3^+^ and CD4^+^CD25^−^Foxp3^−^ are possible source of IL-10 (209). In contrast, IL-10 in human VL is not produced from thymic Foxp3 Tregs; rather they are produced from IFN-γ co-producing CD4^+^ T cells which are called type 1 regulatory (Tr1) cells (143). The role of Tregs was elucidated in modulating both Th1 (210, 211) and Th2 (210, 212) activity during murine *L. major* infection.

### TGF-β Functions Synergistically With IL-10 in Disease Progression

TGF-β is a 28 kDa homodimer and a potent anti-inflammatory cytokine produced by antigen-activated T-cells and mononuclear phagocytes (75). TGF-β has potent immunosuppressive effects in infectious and autoimmune diseases (118), which include inhibition of T-cell proliferation and MΦ activation. TGF-β inhibits the functions of TNF-α and IFN-γ and controls the expression of inducible nitric oxide synthase (iNOS) and the development of Th1 and Th2 response. Unlike IL-10, the impact of TGF-β on parasite burden and IFN-γ dependent host resistance is marginal during *L. donovani* infection (119). Locally activated TGF-β favors the parasite growth by modulating innate and adaptive immune responses (120) and enhancing arginase expression (121, 122). In animal models, TGF-β secreted by *Leishmania* infected lymphocytes diverts the arginine pool from iNOS to arginase for the production of polyamines, which helps in the growth of the parasite (123). Both pro and anti-inflammatory roles of TGF-β have been demonstrated (62, 124). *L. chagasi* infection induces TGF-β secretion by human MΦs through activation of latent TGF-β itself. *L. chagasi* infection also induces the expression of TGF-β in spleen and liver tissues of both symptomatic and asymptomatic dogs (213). TGF-β exposure delays the killing of *Leishmania* parasite and TGF-β overexpression impairs the rate of cure in murine leishmaniasis models (125, 126). In human VL, the elevated levels of IL-10 and TGF-β postively correlate with the parasite load and with increased absolute numbers of FoxP3 Treg cells suggesting the role of Tregs in secretion of these cytokines. There is a significant correlation between the parasite load and circulating antigen specific TGF-β levels in VL patients suggesting its role in parasite multiplication and disease progression in humans (214).

### IL-4 Is Involved in the Pathogenesis of Leishmaniasis but Its Role in VL Is Conflicting

IL-4 is a 20 kDa Th2 subset cytokine that plays a critical role in the regulation of mast cell or eosinophil-mediated immune responses, B-cell mediated IgE class-switching and antibody production. It functions as a growth factor for mast cells and naive CD4^+^ Th2 cells which produce anti-inflammatory cytokines IL-5, IL-10 and IL-13. Both IL-4 and IL-13 inhibit IFN-γ-producing CD4^+^ T-cells and suppress protective Th1 immune response (150) and trigger MΦs to undergo alternative activation resulting in parasite survival and persistence of infection (151, 152). The similarities in IL-4 and IL-13 biological activities are due to a common receptor γ-chain that they both share, which is involved in the signal transduction via signal transducer and activator of transcription (STAT)-6 (215). Studies on murine model established the pathogenic role of IL-4 in leishmaniasis (39). IL-4 inhibits the oxidative burst by inducing low level production of reactive oxygen intermediates and NO in MΦs (153, 154). *L. major* infected Langerhans cells show increased IL-4R expression and decreased IL-12 production in susceptible mice but not in resistant mice (155).

IL-4 modulated antigen-uptake, endosomal processing, and humoral response are suggested to promote the disease development in *Leishmania* infection in humans (156). In murine *L. donovani* infection, IL-4 induces the host protective response (216) and vaccine mediated protection by IFN-γ secretion from CD8^+^ T-cells (157). The peripheral blood mononuclear cells (PBMCs) harvested from cured VL patients produced IFN-γ or IL-4 in response to stimulation by *L. donovani* promastigote or amastigote crude antigen. In response to purified gp63 antigen, the proliferation capability of the same PBMCs was weak and produced IFN-γ or IL-4 (158). Cytokine analysis in VL unveils the induced expression of IL-10 and/or IL-4 mRNA in tissues and abundant levels of IL-4 in circulation of patients with progressive disease (217). Likewise, the conflicting role of IL-4 in VL is described, though it has a leading role in pathogenesis of VL as it is belonging to Th2 phenotype and anti-inflammatory cytokine subset. Recent studies on human splenic aspirates suggest that blockade of IFN-γ and TNF-α results in increased production of IL-4 which does not contribute to parasite replication and IL-10 production. The biological role of IL-4 in target organ of human VL still remains an outstanding question (218).

### IL-13 Promotes Host Protection in VL and Its Role Is Leishmania Species-Specific

IL-13 is a 12-kDa cytokine that is expressed by Th2 and is important in host protection against *Leishmania* infection. For example, IL-13 knock-out mice infected with *L. donovani* show retarded hepatic granuloma formation and maturation, depleted IFN-γ secretion and enhanced production of IL-4 and IL-10 (159). IL-13 protects rats from *L. major* infection through the production of IL-1β and IL-12 (160), which is in contrast to the earlier studies that showed pathogenic role of IL-13 (16, 219). However, studies with BALB/c mice infected by *L. mexicana* and *L. amazonensis* have established the pathogenic impact of IL-4 and IL-13 (161). The susceptibility to *L. (V.) panamensis* infection is predominantly associated with IL-13 but not IL-4. The parasite species and the host genetic background may also influence the dual role of IL-13 and it may not be a potential target for immunotherapy (162).

### Targeting Endogenous IL-6 May Offer Better Protection

IL-6 is a 26 kDa pleiotropic cytokine produced by a number of cell types, including monocytes, endothelial cells and T-lymphocytes (220). The main biological activities associated with IL-6 are the induction of acute-phase protein synthesis in hepatocytes, terminal differentiation of B-cells and activation of T-cells (221). It also induces the production of anti-inflammatory proteins, such as IL-1 receptor antagonist (IL-1rα) and soluble TNF receptor (222). IL-6 plays a major role in the switching of monocytes from DC to MΦs (130). IL-6 favors the development of Th2 response, which suppresses the activation and antimicrobial effect of MΦs (75). Contradicting roles of IL-6 have been demonstrated in experimental CL and VL models (223–225). IL-6 has been shown to either promote (226, 227), suppress (228), or do not change (229) the intracellular host defense to leishmaniasis. Function of endogenous IL-6 as a host suppressive cytokine in case of *L. donovani* infection has outshined its potential pro-host defense effect.

During adoptive transfer of testing splenic DCs, IL-6 induces leishmanistatic effect but not host suppressive effect (226). IL-6 inhibits the IFN-γ mediated gene expression (131) and absence of IL-6 receptor signaling in *L. donovani* liver infection contributes to enhanced Th1-type response, accelerated tissue inflammation, and rapid parasite killing (132). IL-6 induces the secretion of IL-27 which in turn induces IL-10 production (133–135) in the *L. donovani* infected mouse model (54). However, *L. donovani*-infected IL-6^−^/^−^ mice do not show effect on the IL-10 expression (226). This observation raises a potential possibility to target endogenous IL-6 as an anti-leishmanial therapeutic strategy (230). Expansion of CD25^−^FoxP3^−^IL-10^+^CD4^+^ T-cells *in vivo* and therapeutic efficacy of adoptively transferred DCs against *L. donovani* infection are regulated by DC-derived IL-6 (226). IL-6 is produced by dogs with active leishmaniasis and is a key player in the pathogenesis of canine leishmaniasis (144, 231). The presence of TNF-α and IL-6 transcripts was found in both *Leishmania* antigen stimulated and unstimulated cells of asymptomatic infected and uninfected dogs (232). Further, increased anti-leishmanial antibody titers (hypergammaglobulinaemia) in canine VL are usually associated with high levels of IL-6 (224). Contrary to murine VL, the role of IL-6 in human VL is associated with disease severity and death, which is due to the inhibition of TNF-α in the early phase of infection and later by inhibiting the Th1 responses (190, 233).

### IL-27 Contributes to VL Pathogenesis and a Potential Target for Anti-VL Therapy

IL-27 is a member of the IL-6/IL-12 cytokine family and a heterodimer composed of EBI3 and p28. The main cellular source of IL-27 is CD14^+^ spleen cells (140) in particular MΦs and DCs. The anti-inflammatory properties of IL-27 have been demonstrated in various models of infectious diseases and autoimmunity (234). IL-27 mediates anti-inflammatory response by suppressing Th17 cells (138, 139) and inducing IL-10 secretion from activated CD4^+^ T-cells via autocrine action of IL-21 (140). IL-27 plays a multifaceted role characterized by the induction of T-bet (141) in turn inhibition of parasite driven Th2 and Th17 development and Th1 polarization via IL-10 mediated feedback mechanism. IL-27 plays a critical role in the induction of IFN-γ and IL-10 from CD4^+^ T-cells, and suppression of inappropriate Th17 development to achieve immune-balance during intracellular parasite infections. In *L. major* infection, early burst of IL-4 suppresses IL-27 mediated development of normal Th1 by inducing IL-6 and TGF-β and promote the development of Th17 cells (142). In contrast, IL-27 is not required for the normal development of Th1 response to *L. donovani* infection (140) but induces IL-10 production (143, 145).

Absence of IL-27 receptor signaling in *L. donovani* liver infection contributes to the accelerated Th1-type response, tissue inflammation, and rapid parasite killing with reduced parasite burdens in spleen and liver (71, 146). Blocking IL-27 evoke different responses in different mice models. For example, blocking IL-27 results in reduced parasite loads in BALB/c mice and augmented parasite burden is seen in C57BL/6. This dichotomy in the production of IL-27 could be due to the consequence of host immune modulation by the parasite to establish infection (235). IL-27 levels were elevated in human plasma with active VL and splenic mRNA levels of IL-27 and IL-21 were higher in pre-treated biopsies compared with post-treatment samples (140). During VL caused by *L. infantum*, the sequential pathway of TLR3 and TLR9 recruitment, production of type I IFN and activation of IRF1 in macrophages is induced by IL-27. The secretion of IL-27 increases the Th1 response but also dampens the production of IL-17 which directly impacts the reduced recruitment of neutrophils to target organs (236). Inhibition of IL-27 could be targeted for design of anti-VL treatment in the future.

### IL-5 Exerts Moderate Effects on VL Progression

IL-5 is a glycosylated homodimeric 45–60 kDa protein, functions as an anti-inflammatory cytokine and is produced by Th2 cells, mast cells, and eosinophils. IL-5 stimulates the B-cell growth and promotes the production of cytotoxic T-cells from thymocytes; however, the key function of IL-5 is in the activation, maturation, and survival of eosinophils. Eosinophils activated by IL-5 expel antibody bound parasites while releasing proteins associated with cytotoxic granules. In the case of CL and MCL, PBMCs induce secretion of both IL-4 and IL-5 at the site of the lesion (127, 128), which results in declined Th1 polarization. Several studies have reported that IL-4, IL-5, IL-10, and IL-13 provide favorable atmosphere for intracellular parasite growth and dissemination (129). Patients with chronic lesions produce abundant levels of IL-5 and IL-13, which further inhibits parasite killing by an additive effect of IL-13. IL-5 plays a minor role in the susceptibility to *L. major* infection in BALB/c mice (237).

### IL-9 Increases Disease Susceptibility

IL-9 is a 14 kDa pleiotropic cytokine produced by Th-cells, primarily identified as a mouse T-cell growth factor (237) and mast cell differentiation factor. Erythroid precursors, B-lymphocytes, eosinophils, bronchial epithelial cells and neuronal precursors are the secondary targets of IL-9 (238). It is a Th2-type cytokine produced via both IL-4 dependent (239) and IL-4 independent (240) pathways and involved in the physiological regulation of Th1-Th2 balance *in vivo*. Very little is known about the role of IL-9 in leishmaniasis. In *L. major* infection, IL-9 is transiently expressed in susceptible BALB/c as well as in resistant C57BL/6 and DBA mice during early days of infection, but 4-weeks onwards, its expression was only seen in susceptible mice but not in resistant mice (136). *In vivo* neutralization of IL-9 delays disease progression in BALB/c mice by inducing protective Th1 response suggesting, IL-9 promotes susceptibility to *L. major* infection. Further, IL-9 induces classical MΦ activity and production of IFN-γ in *L. major* infected BALB/c mice, which serves as a model system to study the role of IL-9 in human diseases (137).

### IL-33 Is a Prognostic Cytokine for VL Pathogenesis

IL-33 is a member of IL-1 family, which includes IL-1 and IL-18. IL-33 is a crucial player in the defense against nematode infections and allergic reactions, since it causes Th2-type immune response via inducing the production of IL-5 and IL-13 by T-cells, mast cells, basophils, and eosinophils. In addition, IL-33 also induces non-Th2-type inflammation, suggesting its pro-inflammatory role like IL-1 and IL-18. Schmitz et al. first reported that IL-33 functions through ST2 (IL-1R4) orphan receptor present on different immune cell types (241). During *L. major* infection in BALB/c mice, ST2-expressing CD4^+^ T-cells accumulated in local lesions (147). However, administration of polyclonal anti-ST2 antiserum depleted ST2-expressing cells as well as Th2 cells/cytokines and induced Th1 cytokine production, which in turn reduced the lesion development (148). Rostan et al. reported that the serum IL-33 levels were higher in VL patients besides the presence of IL33^+^ cells in liver biopsy of a patient. Similar results were observed in BALB/c mice infected with *L. donovani*, additionally, ST2^+^ cells were also observed in mouse liver. ST2 deficient BALB/c mice had shown strong Th1-type immune response via IFN-γ and IL-12 that controlled the hepatic parasite load and hepatomegaly. Recombinant IL-33 treatment of *L. donovani* infected BALB/c mice dramatically reduced the Th1 immunity and infiltration of polymorphonuclear neutrophils (PMNs) and monocytes in liver. In summary, IL-33 could be a very useful cytokine to determine the host susceptibility and disease prognosis of VL (149).

### IFN-γ Is an Anti-leishmanial Cytokine

IFN-γ is a homodimeric glycoprotein consisting of two subunits each about 21 to 24 kDa. It is the most potent type II interferon that helps in MΦ activation to the leishmanicidal state (63). The main cellular sources of IFN-γ production are activated CD4^+^ and CD8^+^ T-cells, and NK cells in response to IL-12 signaling (242). Of the several anti-leishmanial cytokines (23), IFN-γ is the most significant cytokine in host protection, which plays a prominent role in MΦ priming (64) to produce leishmanicidal molecules (243). IFN-γ acts as monocyte-activating factor (65) and enhances release of oxygen radicals, secretion of pro-inflammatory cytokines (TNF-α, IL-l, and IL-6) (66), expression of MHC class-II, and antigen-presentation. In addition, IFN-γ blocks the production of IL-10, which decreases all the above functions of monocytes (67). Several studies demonstrated that the leishmanicidal activity of MΦs can be induced by a variety of cytokines, either alone or in combination. For instance, lipopolysaccharide (LPS) is required to induce the MΦ leishmanicidal activity *in vitro* (244). In human VL, the response is predominantly Th2-type with absence of PBMCs derived IFN-γ (47). But drug treatment induces a shift in the response so that individuals cured of VL often respond to *Leishmania* antigen by the production of both IFN-γ and IL-4 (158). In CL patients, however, the response is mainly dominated by IFN-γ and IL-4 is rarely detected (245), indicating that the immunological response to *Leishmania* in these individuals does not polarize as observed in inbred mouse strains. *In vitro* studies with T-cell clones (246) and *in vivo* studies using models of CL (40, 43) have demonstrated that IFN-γ can inhibit the expansion of CD4^+^ Th2-cells, resulting in the preferential expression of Th1 cell-mediated response. Reciprocal regulation is provided by the action of IL-10 on the IFN-γ producing capacity of Th1-type CD4^+^ T-cells (16). A recent study showed that the variation in single nucleotide polymorphisms (SNPs) of IFN-γ gene at the position +874 (A/T) influences the susceptibility to VL such that individuals in southwest of Iran with an AT genotype are susceptible and those with a TT genotype are resistant to VL (247).

### IL-12 Is a Promising Candidate for VL Immunotherapy

IL-12 is a heterodimer consisting of two subunits (35 and 40 kDa) linked by a disulfide bond, mainly produced by activated MΦs and DCs. It is a proinflammatory cytokine that plays a key role in bridging innate and adaptive immune responses (248). Protective immunity against leishmaniasis is typically associated with the production of IL-12 (16, 219). IL-12 drives Th1 response and induces IFN-γ production from both NK cells and T-cells (68), and mediates the leishmanicidal activity by inducing NOS2 expression and NO production (146). In addition, IL-12 also mediates T-cell proliferation and lymphokines production (71, 72). The presence of IL-12 reduces the ability of CD4^+^ T-cells to produce IL-4 and increases the ability to produce IFN-γ. Thus, IL-12 and NK cells seem to play important role in determining the development of Th1 response (249). *In vivo* studies showed that IL-12 produced in infected mice (219, 250) is important to control Th2 expansion and to promote Th1 type response (69, 70). Neutralization of IL-12 leads to disease exacerbation in *L. major* and *L. donovani* infections (49, 69, 77). In contrast, the addition of IL-12 to lymphocyte cultures from VL patients restored IFN-γ production and increased cytotoxic activity of NK cells (48).

### Endogenous TNF-α Offers Protection in VL

TNF-α is a 51 kDa homodimeric cytokine, mainly secreted by the activated MΦs, T-cells, NK cells and mast cells. TNF-α is important in mediating both innate and adaptive inflammatory responses. The regulation of TNF-α production appears to be important because it has potential role in the formation and maintenance of granuloma (76). Antiparasitic activity of TNF-α is mediated through activation of infected MΦs for the destruction of intracellular amastigotes (73). TNF-α production is absent in susceptible mice but present in *L. major* infected resistant mice. Protective role of TNF-α in *L. major* infection is characterized by MΦ activation, NO production and parasite clearance or suppression of visceralization (74). Protective T-cell response induced by TNF-α in *L. major* infected mice is due to the induced production of parasite-specific IgG1 and IgG2a. Acute infection with *L. braziliensis* resulted in the lack of production of parasite-specific IgG1 and IgG2a antibodies (251). The role of TNF-α in *L. braziliensis* infection is attributed to controlling the parasite numbers in the skin, lymph nodes and spleen and wound healing process (75). Brazilian patients with MCL had increased levels of TNF-α in both sera (252) and tissue lesions (253).

Treatment with TNF inhibitors, such as pentoxifylline in combination with anti-leishmanial pentavalent antimony, pentoxifylline promotes the re-epithelialization of mucosal tissues (254). However, infection of TNF^−^/^−^ mice with *L. major* shows some delay but no defect in antigen-dependent T-cell activation (74). IFN-γ independent anti-leishmanial mechanism mediated by endogenous TNF-α was described in IFN-γ knockout (GKO)-1 mouse infected with *L. donovani* (77, 199). The *L. donovani* infection provoked endogenous TNF-α level are enough to offer initial resistance to the parasite invasion and critical for the resolution of visceral infection. This is contrasting with the effect of exogenous TNF-α, which has no protective role in established infection and its continuous administration leads to impaired anti-leishmanial activity (255). The polymorphism and upregulation of TNF2 promoter transcription could be involved in enhancing clinical VL infection (256). TNF-α cannot be considered as a good marker of active disease in both human VL and canine VL due to its labile nature (224). The production of TNF-α follows biphasic kinetics due to its effect on target cells mediated by membrane-bound receptors (117). The high expression of IL-32 (especially γ-isoform) in CL and mucosal lesions is associated with endogenous TNF-α production but not with IL-10, suggesting the inflammatory role of IL-32 in host defense against *Leishmania* infection (257). Absence of IL-32 leads to high infection index but its overexpression opposed the parasite growth via NO cathelicidin and β-defensin 2 syntheses (258). In response to excessive inflammation, increase in the levels of TNF-α might promote the generation of IL-10 producing T-cells as a homeostatic response (78).

### IL-2 Promotes Anti-leishmanial T-Cell Response

IL-2 is a 15 kDa cytokine, produced by activated T-cells and was initially identified as a T-cell growth factor. IL-2 stimulates the proliferation and differentiation of B-cells, NK cells, monocyte/MΦs, oligodendrocytes and lymphocyte activated killer (LAK) cells. IL-2 does not directly stimulate the intracellular antimicrobial activity of MΦs (259) but exerts a range of immunoregulatory effects on T-cells and NK cells and induces the production of IFN-γ (79, 80). IL-2 may act as a susceptibility factor in leishmaniasis (250, 260) by inducing the production of IL-4 from CD4^+^ T-cells (81). But in IL-4 deficiency, the inhibition of disease progression is attributed to IL-13 and IL-2. Interestingly, in CL, both IL-2 and IL-15 are attributed in host protection, while in MCL IL-2 is only protective but not IL-15 (200). Rapid production of IL-2 was observed after successful treatment or acquisition of resistance against *L. donovani* infection but not during acute phase (261, 262). The endogenous IL-2 could act as a defensive cytokine, only when the mice subsequently challenged after a prior infection with the parasite (82). In contrast, exogenous IL-2 exerts the anti-leishmanial action using L3T4^+^ and Lyt-2^+^ T-cells in acutely infected euthymic mice. IL-2 exerts leishmanicidal activity in splenocytes *in vitro* even in the absence of IFN-γ (83).

### IL-15 Synergizes IL-2 and IL-12 in Host Defense and Has Scope in VL Therapy

IL-15 is a 14–15 kDa cytokine with four α-helix bundles and plays a central role in the innate and adaptive immune responses to infections (86). The main source of IL-15 is activated peripheral monocytes (263). Due to the common receptor β-chain, immunological functions of IL-15 are similar to IL-2 (84), which includes the induction of T-cell proliferation (87), inhibition of T-cell apoptosis and preservation of memory T-cells (88), B-cell maturation and isotype switching (264). IL-15 also stimulates the proliferation and activation of NK cell (265) and induces the production of IFN-γ and TNF-α, synergistically with IL-12 (85). Nevertheless, IL-15 also shows distinct biological functions from IL-2 due to a different α-chain (266, 267). The possible pleiotropic role of IL-15 is reflected by its action on both Th1 and Th2 subtypes and the ability to induce the activity of IFN-γ and IL-12 (89) as well as IL-5 and IL-13 (90) in various experimental models. Endogenous IL-15 stimulates protective Th1 response by inducing the downregulation of IL-4^+^ Th2 cells (86). IL-15 could be a potential therapeutic agent in acute VL since it upregulates IL-12 production and Th1 development (91). In contrast, other studies have demonstrated that endogenous IL-15 is not necessary for basal expression of IL-12 and MΦ activation and is not able to influence the IL-12 activity and Th1 development in acute VL (268). IL-15 in combination with IFN-γ and/or IL-12 may increase the efficacy of conventional antimonial therapy for VL, because of low toxicity *in vivo* (201).

### IL-17 Role Is Contradictory in Leishmaniasis

IL-17 is a 35 kDa proinflammatory cytokine, primarily produced by activated T-cells (CD4^+^ > CD8^+^) (269) and also by other subsets of T-cells including NKT cells and Th17 cells (96). The development of Th17 subset from naïve T-cells happens in the presence of IL-6 and TGF-β^+^ Tregs (270). IL-17 stimulates different immune cells to produce inflammatory molecules including TNF-α, IL-1, and chemokines (163). At the site of inflammation, IL-17 affects the neutrophil function, reduces the apoptosis, and promotes the secretion of pro-inflammatory cytokines as well as tissue damaging molecules (164, 165). IL-17 induces the secretion of granulocyte macrophage-colony stimulating factor (GM-CSF) and G-CSF, which increase the production of neutrophils, monocytes, and chemoattractants for neutrophils (CXCL8, CXCL1, and CXCL6) as well as Th1 cells (CXCL10) (96, 97). IL-17 induces the production of IL-6, which mediates both proinflammatory and regulatory functions (96). IL-17 of Th17 subset and Th1 subset play a complementary role in the host protection from *L. donovani* infection. Contrastingly, the susceptibility for *L. major* infection is not only associated with uncontrolled Th2 immunity (271) but also with excessive IL-17 secretion, which mediates neutrophil recruitment (166). Other studies have also demonstrated that IL-17 dependent neutrophil recruitment is essential only during the late stages but not early stages of *L. major* infection (272). Mucosal disease caused by *L. (V.) braziliensis* is also associated with elevated levels of IL-17 response (167). Treating VL using curdlan, a b-glucan immunomodulatory molecule induces Th1 cytokines with IL-12, IL-22 and IL-23 (273), while another immunomodulator Astrakurkurone is effective against experimental VL by inducing IL-17 along with IFN-γ (274). While these reports are suggestive of a protective role of IL-17 in VL, other reports suggested the involvement of IL-17 in exacerbating experimental VL in murine model (275) raising questions about its precise role in VL pathogenesis.

### IL-18 Protects From VL but Favors Other Forms of Leishmaniasis

IL-18 is a 22 kDa pleiotropic cytokine produced by activated MΦs and Kupffer cells of liver (276). IL-18 promotes Th1 and NK cell development (168), induces IFN-γ production by lymphocytes and NK cells and synergizes with IL-12 (169, 170). IL-18R is expressed on Th1 cells but not on Th2 cells. IL-18 induced Th1 subset produces IFN-γ which indirectly regulates the expansion of Th2 cell (171). IL-18 promotes NK cell activity due to a constitutive expression of IL-18R on NK cells (277) and stimulates TNF-α secretion by human PBMCs (172). IL-18 also induces the activation of memory cells and in combination with IL-12 it prevents the reinfection of BALB/c mice with *L. major* (173). IL-18^−^/^−^ mice are highly susceptible to *L. donovani* infection when compared to the wild-type mice. However, endogenous IL-18 induces an initial IFN-γ independent anti-leishmanial effect in *L. donovani* infection (174). Paradoxically, IL-18 can also stimulate Th2 cytokines production, such as IL-4 from basophils (175) and CD4^+^ T-cells (176) and IL-13 from mast cells. While IL-18 protects BALB/c mice from *L. donovani* infection, it increases the susceptibility of BALB/c mice to *L. major* infection. Notably, the resistance and susceptibility of BALB/c mice to *L. mexicana* infection are not mediated by IL-18 and are influenced by different genetic and immunoregulatory controls (278). IL-18^−^/^−^ BALB/c mice are highly resistant to *L. mexicana* infection due to increased IFN-γ production and antigen-specific IgG2a, reduced splenic IL-4, antigen-specific IgG1 and total IgE (177). IL-18 plays a critical role in the regulation of Th1 and Th2 balance *in vivo*, which frequently determines the outcome of many important infectious and autoimmune diseases (168).

### IL-22 Offers Protection From VL

IL-22 is a 16.7 kDa cytokine, primarily produced by Th17 cells and to a lesser extent by Th1 and NK cells (96). Immunological functions of IL-22 are associated with the epithelium and mucosal surfaces (92), which include promoting inflammatory response and tissue repair (93, 279). IL-22 stimulates the production of pro-inflammatory molecules, such as S-100A proteins and CXCL5 (93). IL-17 and IL-22 act synergistically on epithelial cells to produce an antimicrobial peptide called β-defensin (95). IL-22 is also involved in protecting the liver (94) during chronic infections. During *L. donovani* infection, both IL-17 and IL-22 are produced by PBMCs and may exert complementary function along with Th1 cytokines (96, 97). The production of IL-22 requires IL-6 but not TGF-β (98).

### Role of IL-1 Is Protective in VL but Contradictory in Other Forms of Leishmaniasis

IL-1 is synthesized as ~35 kDa precursor, from which two functional agonistic proteins (IL-1-α and IL-1-β each 17 kDa M.W.) and IL-1Ra, receptor antagonist of IL-1R1, are produced. IL-1 is a potent proinflammatory “alarm cytokine” that synergizes the functions of TNF-α and is produced by MΦs. IL-1 builds inflammation by inducing the expression of adhesion molecules on endothelial cells and leukocytes (280, 281). IL-1β, along with other proinflammatory cytokines, is released into the periphery during infection and coordinates immune-to-brain communication (180). IL-1 mediated inflammation is coordinated by adaptive T-cell response and controls the parasite dissemination (178, 179). IL-1 is responsible for regulating the delicate balance between inflammation and immunity which decides the fate of the disease progression in leishmaniasis. In *L. major* infection, the acute levels of IL-1α, IL-1β, and IL-1Ra are adequately downregulated unlike in *L. amazonensis* infection (282). Disease progression is inhibited with IL-1α treatment in *L. major* susceptible BALB/c mice during T-cell differentiation. IL-1β enhances activation of DCs and T-cell priming but do not affect the cytokine profile of DCs and pathogenic Th-cells (178). Contrastingly, Voronov et al. reported that BALB/c mice deficient in IL-1 family genes showed delayed disease progression with *L. major* infection due to apparent Th1 response even at late stages of the disease. IL-1α deficient mice were slightly more resistant to *L. major* infection than IL-1β KO mice (181). In *L. amazonensis* infection, IL-1β treatment induced DCs and cytokine production remains lower than that of *L. major* infection. IL-1 therapy in murine CL results in a wide range of outcomes depending on the course of treatment and parasite species involved. In this context, IL-1-based treatment may be effective for *L. major* but not *L. amazonensis* infection. The decreased production of IL-1 has been associated with *L. donovani* infection of murine peritoneal MΦs *in vitro* and human circulatory monocyte population (182, 183). Similarly, human PBMCs failed to produce IL-1 in response to *Leishmania*-antigen stimulation *in vitro*, during acute VL. However, following anti-leishmanial therapy, IL-1 and TNF-α levels are usually recovered, which correlates with clinical cure (184). Recombinant IL-1α induces mature granuloma formation in liver and IFN-γ production from spleen cells but is not able to clear the parasite (185).

### IL-3 Is Likely a Host Protective Cytokine in VL

IL-3 is a 28 kDa glycoprotein derived from T-cells and supports the viability and differentiation of hematopoietic progenitor cells (283, 284) and monocytes (283). With the combination of GM-CSF, M-CSF, and IFN-γ, IL-3 shows an additive effect on human MΦs in the induction of oxidative burst and TNF-α secretion to inhibit the replication and growth of *Leishmania* parasite. In contrast, IL-3 promotes the infection in murine model of CL highlighting the species-specific differences in the role of IL-3 in leishmaniasis (186). In combination with M-CSF, IL-3 induces superoxide ions production to kill the parasite and may involve in myelopoiesis during acute VL.

### IL-7 Shows Additive Effect With IFN-γ Against Leishmania

IL-7 is a 17 kDa glycoprotein derived from bone marrow stromal cells (285) and regulates a wide variety of functions including multiple effects on B-cells and proliferation of thymocytes (99–101), NK cells (102) and mature T-cells (103). IL-7 induces the production of cytotoxic T-cells with alloreactive, antitumor, and antiviral activities (104). It was reported that IL-7 shows potential effects on monocytic lineages (105, 286). IL-7 enhances the synthesis and secretion of various inflammatory cytokines, such as IL-6, TNF-α, IL-1α, IL-1β, and MΦ inflammatory protein (MIP) 113 by human circulatory monocytes. IL-7 is not as effective as IFN-γ but shows an additive effect with the combination of suboptimal concentrations of IFN-γ in killing the *Leishmania* amastigotes by inducing the production of TNF-α (105) and NO.

### IL-8 Attracts Neutrophils to the Site of Infection

IL-8 is a non-glycosylated proinflammatory cytokine with a M.W. of 8 kDa, which is primarily produced from neutrophils and from other cell types including epithelial cells, keratinocytes, fibroblasts and endothelial cells. In mouse system, IL-8 has two functional homologs like MIP-2 (CXCL2/Groβ) and KC (CXCL1/Groα). During *L. major* infection in humans, IL-8 promotes the recruitment of neutrophils at lesion sites (106). In mice infected with *L. major*, IL-8 causes transient production of KC mRNA in the skin, which may associate with granulocyte recruitment (107) which is yet to be demonstrated *in vivo* (272). Notably, a reduced neutrophil count during active VL is associated with lower IL-8 levels in serum (32). It was identified that polymorphisms at IL-8 −251 position are associated with impaired IL-8 activity and the development of active VL in Iranian individuals (108) but such a correlation was not observed in Brazilian VL patients (287).

### IL-23 May Offer Protection From VL in Association With IL-12p40

IL-23 is a pleiotropic cytokine produced by MΦs and DCs which acts on receptors expressed by T-cells, NK cells and NKT cells (288). During *L. donovani* infection in BALB/c mice, the IL-23p19 mRNA expression in the liver tissue was comparable to that of wild-type. IL-12 independent protection in visceral infection (76, 109, 110) was mediated by IL-18 and probably by IL-23 also, since the p40 subunit of IL-12 shared by both IL-12p70 and IL-23 (174). IL-23p19 may pair with IL-12p40 to become biologically active (111), which are crucial for host protection. Therefore, IL-23 alone or in combination with other cytokines may be a possible option in immunotherapy of VL.

## Interplay of T-Cell Subsets via Cytokines in Leishmaniasis

It is well established that the complex interplay of pathogens with their hosts is predominantly regulated by host-specific Th1/Th2 subset cytokines in the vicinity of several regulatory cytokines. In this context, previous studies have demonstrated that IL-10 produced by CD4^+^CD25^+^ Tregs (211) is important for parasite persistence in mice (289). In human VL, the elevated level of IFN-γ mRNA in lymphoid organs (spleen and bone marrow) is accompanied by an abundant expression of IL-10 (55, 290, 291) where the predominant source of IL-10 is Foxp3^−^CD25^−^CD3^+^ cells (143). However, in leishmaniasis, healing is predominantly associated with diminished expression of IL-10 mRNA (55, 290). The role of Th17 subset in human VL is unveiled by a longitudinal study in Sudan, which illustrated the protective role of Th17 subset that are employed by an induced production of IL-17 and IL-22 from *L. donovani*-specific T-cells (292). In fact, Th17 cells are pleiotropic in nature, responsible for either protection or pathogenesis and frequently associated with recruitment of neutrophils. Th17 cells under the influence of IL-27 producing CD4^+^ T-cells diminish IL-17 and IL-22 secretion (293). Disease progression in pre or post-treated Indian VL patients is linked with serum IL-27 and splenic IL-27 transcript but not with splenic IL-17 transcript (140).

The pathogenic role of IL-27 in active VL is linked with suppression of Th17 cytokines production and expression of transcription factors. Consequently, IL-27 promotes the parasite dissemination by inducing antigen-specific IL-10^+^ T-cell differentiation and expansion, and by inhibiting activation of effector Th17 lineage. Moreover, the host negotiates the Th17 response to control the pathogenic implications of VL that are driven by the parasite. Th9 cells are not the unique source of IL-9 production as Tregs and Th17 cells also produce IL-9 in lesser quantity. Initially, Th9 subset was thought of a splinter group of Th2 but now it is an independent lineage. Predictably, Th9 subset has a similar detrimental role like Th2 in the development of CL in the mouse model. Since, IL-4, IL-21, TGF-β, and IFN-α/β seem to activate Th9 cells to produce IL-4, IL-9, and IL10, which are involved in the pathogenesis of CL (136, 137). The “B-helper” follicular T-cell (Tfh) lineage is also implicated in leishmaniasis progression, which is the source for bulk production of IL-4 in the draining lymph nodes of susceptible mice infected by *L. major* (294). As a sequel of VL, PKDL patients' carries high plasma IL-10 levels (295). Immunologically, PKDL is characterized by hyper T-cell response and significant production of both Th1 and Th2 cytokines from PBMCs in response to crude *L*. *dono*v*ani* antigen (296). IL-10 levels in the skin and plasma could be used to predict the severity of PKDL pathogenesis and the chance of VL succession to PKDL.

## Cytokines in VL Diagnosis and Immunotherapy

As immunotherapy is mandatory for refractive cases of leishmaniasis, cytokines received great attention in the search for novel approaches for the diagnosis and immunotherapy of VL (summarized in Table 4). For the first time, Reed et al. used the lymphokines obtained from murine spleen cell culture supernatant and encapsulated in liposomes against VL challenge and reported a significant reduction in the liver parasite burden, highlighting the importance of lymphokines in leishmaniasis healing (297). In general, the absence of leishmanial-antigen stimulated lymphocyte proliferation and IFN-γ production are indicators for the clinical evaluation of a VL patient (45). However, these two parameters may also be used as coordinates in assessing the level of protection conferred by vaccine antigens (194). Later, several studies have tested the effect of direct administration of recombinant Th1 cytokines and monoclonal antibodies against Th2 cytokines alone or in combination against leishmaniasis. For example, prophylactic anti-IL-10R treatment induces the rapid control of experimental VL and antimonials activity in IL-10 knock-out or transgenic mice (51), and IFN-γ production from T-cells with an active VL (55). Further, it was reported that IL-10R inhibition in *L. donovani*-infected mice controlled the parasite burden in liver, increased IFN-γ titers in serum, and iNOS production in macrophages altogether reduced the VL fatality (298). The therapeutic efficacy of anti-IL-10R and anti-GITR (glucocorticoid-induced TNF receptor-related protein) was tested against *L. donovani* challenge in C57BL/6 mice. Blocking IL-10 alone could reduce the parasite burden in spleen and liver but combination of these antibodies did not inhibit the parasite proliferation in spite of the significant increase in IFN-γ and TNF-α production (299). In another study, the blockade of IL-2 and IL-10 was effective in the reduction of parasite load in early and later phases of *L. donovani* infection in BALB/c mice (300). IL-10 neutralization in splenic aspirate cells increases IFN-γ and TNF-α production and reduces parasite burden in VL patients (187). In *L. chagasi* infected Brazilian population, the antigen-stimulated PBMCs derived IL-10 titers were higher in acute VL than after cure. However, Leishman skin test (LST) or Montenegro test positivity is not directly correlated with the IL-10 production in asymptomatic individuals (113). The balance between IL-10 and IL-12 determines the effectiveness of chemotherapy (17).

IL-4 induced in VL is usually associated with impaired treatment (191, 192). IFN-γ, IL-4, and IL-13 are upregulated in active VL, however, their levels are significantly declined after cure (193). The disease relapse in human VL patients is associated mostly with IL-10 rather than IL-13 and is influenced by IL-10^+^ IFN-γ^+^ antigen-specific T-cells (189). Blocking IL-4, IL-13, and TGF-β with receptor fusion antagonists substantially controlled the parasite replication but the clearance of visceral infection is marginal and had no synergistic effect with Sb^V^ (119). Though IFN-γ, IL-6, IL-27, TNF-α, and IL-10 levels increased in Brazilian patients with active VL caused by *L. infantum*, the clinical manifestation are strongly correlated with IL-6, IL-27, TNF-α, and IL-10 (190). In general, TNF-α synergizes IFN-γ in the activation of MΦs and clearance of parasite but it is found to elevate in serum despite the low TNF-α^+^ monocytes in the circulation of active VL patients (301). To surpass the side-effects, rIFN-γ and muramyl tripeptide (MTP-PE) encapsulated in liposomes at varying doses of intravenous (i.v.) injections causes substantial reduction in the splenic parasite burden in murine VL (302). rIFN-γ was tested in combination with Sb^V^ against VL patients from Brazil, Kenya, and India and the therapeutic efficacy was found to be 82.3, 75, and 87%, respectively (195–197).

IL-12 orchestrates acquired resistance in liver during intracellular *L. donovani* infection and parasite killing (174). IL-12 restored responses from PBMCs of VL patients much better than the treatment with anti-IL-10 alone or in combination with anti-IL-4 [53]. Hence, it is clear that successful VL therapy is associated with restoration of IFN-γ and IL-12 production (47). IL-12 was used as an effective adjuvant for a killed vaccine against *L. major* (198). The treatment of susceptible BALB/c mice with recombinant IL-12 mediates disease healing, which is associated with induced production of Th1 cytokines (81, 200) and suppression of IL-4 (75). The treatment for arthritis with anti-TNF-α results in increased susceptibility to VL (202, 203), suggesting that TNF-α could act as a primary effector component. Upon liposomal amphotericin B treatment, the plasma IL-15 levels were found to be increased in VL patients (86), suggesting the role of IL-15 as a marker in VL diagnosis and a target in the VL therapy. In opportunistic HIV co-infection, high levels of serum TNF-α and IFN-γ are the predictors for onset of acute VL infection (204). Recently, we demonstrated cytokine role and therapeutic potential of recombinant leptin (adipokine) in BALB/c mice with experimental VL caused by *L. donovani*. The serum leptin levels and splenic Th1 cytokine response were found to be reduced in active disease. Upon leptin administration, host protective responses including Graz-A^+^ CD8^+^ T-cells, IFN-γ, IL-12, and IL-2 production were found to be restored (303). Hence, low systemic leptin levels could be of prognostic and diagnostic value in the assessment of clinical VL.

## Limitations and Future Prospective

Although cytokine-based immunotherapy is a promising approach for VL cure, there are certain limitations associated with this strategy. The production of recombinant cytokines as large molecules used in therapeutics is very expensive and they must be administrated via injections, which is certainly painful to the patients. Administering high dose of cytokines could result in side effects characterized by malaise and influenza-like syndromes. As the cytokines have short half-life in plasma, multiple doses are need which further increases the side-effects (304). A crucial aspect is that the cytokine therapy for leishmaniasis must be cost-effective over conventional treatment in order to be practical. It is possible that a combination therapy comprising a potent anti-leishmanial cytokine with the combination of an inhibitor (monoclonal antibody) targeting disease promoting cytokine or with current drug options could be a future prospective of leishmaniasis treatment. However, there is possibility that the different combinations of cytokines may produce a divergent immune response. Hence, it is important to further investigate the effect on immune response to develop a clinically relevant combination therapy. This is particularly important since several cytokines share common signaling cascades as outlined in this review, which affects the outcome of treatment. The gene manipulation strategy using advanced molecular biology tools may produce desired version of cytokines with small ligand-tags that have the potential to increase the half-life of cytokines from minutes to days in the blood by tethering with albumin protein, which further could reduce the number of required doses. After finding a successful combination of these against leishmaniasis, it would be optimal to design the chimeras of cytokines without losing native structural and functional properties. After administration, the chimeric cytokines should splice inside the body fluids and act independently. As a second option, cytokine and drug combination was also shown to be a reliable strategy against leishmaniasis. For example, combination of IFN-γ with antimony against experimental VL showed that antimony dosage required for leishmanicidal activity was reduced by 4- to 10-folds with IFN-γ combination (305). This is a string indication that administering a drug-cytokine mix could address the drug toxicity and possible development of resistance. In another study, the pre-treatment for 20 days with IFN-γ before antimony therapy has cured the VL in 4 out of 9 Indian patients and rest of them had shown reduced parasitemia in spleen aspirates (197). As mentioned earlier, CTLA-4 and PD-1 causes T-cell unresponsiveness, so targeting these for leishmaniasis treatment may yield promising results. A study showed that the anti-CD40 and anti-CTLA-4 with the combination of Sb^V^ against *L. donovani* infection in a mouse model increased IL-12 and IFN-γ production, T-cell activation and function, and synergistic with Sb^V^ while increasing the parasite death (306). Similarly, administration of chimeric fusion protein OX-40L-Fc and anti-CTLA-4 improved granuloma maturation and CD4^+^ T-cell proliferation to augment the killing *L. donovani* parasite but had no effect on IL-10 and TGF-β production (307). Liposomal amphotercin B treatment with the combination of recombinant human granulocyte macrophage colony-stimulating factor (rHuGM-CSF) cured the VL clinical symptoms and splenomegaly in a patient suffering from HIV and VL (308). Another promising therapeutic option is administration of anti-leishmanial drugs and immunomodulators together. For example, the suboptimal doses of miltefosine with the combination of a single dose of TLR-ligand called Pam3Cys (tripalmytoil-Cysteine), an immunomodulator, significantly promoted the healing of *L. donovani* infection in mice by increasing the production of Th1/Th2 cytokines, reactive oxygen and nitrogen intermediates, and H_2_O_2_ (309). Cytokine producing immune cell-based therapy either alone or in combination with drugs has recently emerged as a potential treatment for cancer and other infectious diseases. Glycosphingophospholipid (GSPL), a β-(1–4)-galactose terminal NKT-cell ligand of *L. donovani* antigen induces inflammatory signaling cascade to kill the intracellular parasite, induces effector T-cell response and controls the acute parasite load to an undetectable level in experimental VL (310). DCs could also be an attractive option as they are important antigen-presenting cells at the interface of innate and acquired immunity and can suppress early dissemination of the parasite to the lymphoid tissues mediated by IL-10 (311). Combination of bone marrow-derived DCs pulsed with *L. donovani* antigen and antimony treatment completely cleared the infection from the spleen and liver (312) by inducing Th1 cytokines production (313). Cytokines are the key players in the determination of disease outcome during various immunotherapies. It is important to remember that measuring the levels of a pro- or anti-inflammatory cytokine alone to predict the disease severity may not be reliable. Measuring the ratio of cytokines is a promising approach. For example, IFN-γ/IL-10 ratio is predictive of disease severity in VL (314).

## Conclusive Remarks

As the cytokines are the key focus of various immunotherapies against leishmaniasis, it is essential to understand their role in detail with possible scope in developing novel diagnostics and targeted therapy for VL. There are key set of cytokines that are involved in the disease progression namely IL-10, TGF-β, and IL-4 and host protection namely IFN-γ, IL-12, TNF-α, and IL-2 during VL. Notably, there are other cytokines that are also involved in the pathogenesis and host defense during VL. However, their role appears to be complex and is dependent on the *Leishmania* species and the type of clinical disease. For example, cytokines namely, IL-1, IL-13, IL-17, and IL-18 are involved in the host defense during VL but have an opposite effect by promoting the disease in CL. Nonetheless, cytokines involved in the host protection e.g., IL-15, IL-22, and IL-23 and pathogenesis e.g., IL-33, IL-27, IL-9, and IL-21 can be explored further as promising targets in diagnosis and immunotherapy of VL.

## Author Contributions

Topic selection and content development was done by AD and SKK. First draft was prepared by AD and SKK. SVK have corrected and revised to final version. SC has been supportive in images and tables.

### Conflict of Interest Statement

The authors declare that the research was conducted in the absence of any commercial or financial relationships that could be construed as a potential conflict of interest.
